# Quadrivalent meningococcal tetanus toxoid-conjugate booster vaccination in adolescents and adults: phase III randomized study

**DOI:** 10.1038/s41390-023-02478-5

**Published:** 2023-03-10

**Authors:** Betzana Zambrano, James Peterson, Carmen Deseda, Katie Julien, Craig A. Spiegel, Clifford Seyler, Michael Simon, Robert Hoki, Marc Anderson, Brad Brabec, Germán Áñez, Jiayuan Shi, Judy Pan, Audrey Hagenbach, Dalia Von Barbier, Kucku Varghese, Emilia Jordanov, Mandeep Singh Dhingra

**Affiliations:** 1 Global Clinical Development Strategy, Sanofi, Montevideo, Uruguay; 2 J. Lewis Research, Salt Lake City, UT USA; 3 Caribbean Travel Medicine Clinic, San Juan, Puerto Rico; 4 J. Lewis Research Inc, South Jordan, UT USA; 5 Craig A. Spiegel, MD Bridgeton, Bridgeton, MO USA; 6 Pediatric Clinical Trials Tullahoma, Tullahoma, TN USA; 7 Michael W. Simon, MD, PSC, Lexington, KY USA; 8 Wee Care Pediatrics, Layton, UT USA; 9 Tanner Clinic, Layton, UT USA; 10 Midwest Children’s Health Research Institute, Lincoln, NE USA; 11 Global Biostatistical Sciences, Sanofi, Swiftwater, PA USA; 12 Sanofi, Marcy L’Étoile, France; 13 Sanofi, Swiftwater, PA USA; 14 Present Address: Global Clinical Development Strategy, Sanofi, Swiftwater, PA USA; 15 Present Address: Global Clinical Immunology, Sanofi, Swiftwater, PA USA

## Abstract

**Background:**

The immunogenicity and safety of a booster dose of tetanus toxoid-conjugate quadrivalent meningococcal vaccine (MenACYW-TT), alone or co-administered with MenB vaccine, were assessed in healthy 13–25-year olds who received MenACYW-TT or a CRM-conjugate vaccine (MCV4-CRM) 3–6 years earlier.

**Methods:**

This phase IIIb open-label trial (NCT04084769) evaluated MenACYW-TT-primed participants, randomized to receive MenACYW-TT alone or with a MenB vaccine, and MCV4-CRM-primed participants who received MenACYW-TT alone. Functional antibodies against serogroups A, C, W and Y were measured using human complement serum bactericidal antibody assay (hSBA). The primary endpoint was vaccine seroresponse (post-vaccination titers ≥1:16 if pre-vaccination titers <1:8; or a ≥4-fold increase if pre-vaccination titers ≥1:8) 30 days post booster. Safety was evaluated throughout the study.

**Results:**

The persistence of the immune response following primary vaccination with MenACYW-TT was demonstrated. Seroresponse after MenACYW-TT booster was high regardless of priming vaccine (serogroup A: 94.8% vs 93.2%; C: 97.1% vs 98.9%; W: 97.7% vs 98.9%; and Y; 98.9% vs 100% for MenACWY-TT-primed and MCV4-CRM-primed groups, respectively). Co-administration with MenB vaccines did not affect MenACWY-TT immunogenicity. No vaccine-related serious adverse events were reported.

**Conclusions:**

MenACYW-TT booster induced robust immunogenicity against all serogroups, regardless of the primary vaccine received, and had an acceptable safety profile.

**Impact:**

- A booster dose of MenACYW-TT induces robust immune responses in children and adolescents primed with MenACYW-TT or another MCV4 (MCV4-DT or MCV4-CRM), respectively.
- Here, we demonstrate that MenACYW-TT booster 3–6 years after primary vaccination induced robust immunogenicity against all serogroups, regardless of the priming vaccine (MenACWY-TT or MCV4-CRM), and was well tolerated.
- Persistence of the immune response following previous primary vaccination with MenACYW-TT was demonstrated.
- MenACYW-TT booster with MenB vaccine co-administration did not affect MenACWY-TT immunogenicity and was well tolerated.
- These findings will facilitate the provision of broader protection against IMD particularly in higher-risk groups such as adolescents.

## Introduction

Invasive meningococcal disease (IMD) is a life-threatening illness caused by the obligate human bacterium, *Neisseria meningitidis*. IMD is a leading cause of mortality and morbidity globally, with a fatality rate of 8–15%.^1^ It is often associated with long-term sequelae amongst survivors, such as neurological complications, hearing loss, loss of limbs and paralysis.^2^ The incidence of IMD peaks in infants under 1 year of age with a second smaller peak in incidence observed in adolescents and young adults in many countries.^3^ Notably, carriage rates have been observed to be highest in this latter age group.^4^

Of the 12 meningococcal serogroups identified, 6 (A, B, C, W, Y and X) are the causative agent in the majority of cases of IMD worldwide.^5,6^ The dominant serogroup varies by geographical region and fluctuates unpredictably over time. In Western Europe and Canada, meningococcal serogroup C (MenC) outbreaks in the late 1990/early 2000s led to a steep rise in the incidence of IMD, while an increased incidence of MenW has been more recently observed in these countries.^7^ In the US, most outbreaks over the past two decades have also been caused by C and more recently B, with a smaller proportion of IMD cases caused by Y and W.^8^ Vaccination campaigns with monovalent (MenC) and quadrivalent (MenACWY) polysaccharide-protein conjugate vaccines have successfully reduced IMD incidence in many countries where this has been implemented.^7–12^ The recent development of two novel recombinant protein vaccines against MenB offers additional protection against IMD caused by serogroup B.

In the United States, the Advisory Committee on Immunization Practices (ACIP) recommends routine primary vaccination with a meningococcal conjugate vaccine (MCV4) in children aged 11 or 12 years, and a booster dose at age 16.^13^ Several European countries also recommend MCV4 vaccination in toddlers and/or adolescents.^14^ Since their launch in 2015, MenB vaccines have been introduced into routine national vaccination programs, often in infants, in a number of European countries, and other countries worldwide including the UK, US and Australia.^14–17^

MenACYW-TT (MenQuadfi®; Sanofi, Swiftwater, US) is a quadrivalent meningococcal polysaccharide (Serogroups A, C, W and Y) tetanus toxoid-conjugate vaccine, and does not contain an adjuvant. It was licensed in the US for use in individuals aged ≥2 years of age, and individuals aged ≥12 months in the EU, Australia, Canada, Brazil, and other countries. MenACYW-TT has been extensively evaluated in toddlers, children, adolescents and adults (including those over 65 years),^18–21^ and more recently in infants from 6 weeks of age.^22^ A dose of MenACYW-TT in those primed 4–10 years previously with an MCV4 (MCV4-DT or MCV4-CRM), at age 10 years or older, was previously demonstrated to boost the immune response in adolescents and adults.^23^ Similarly, a dose of MenACYW-TT in children aged 4–5 years who received a primary dose of MenACYW-TT or MCV4-TT as toddlers also demonstrated robust boosting of the immune response.^24^

The aim of this phase IIIb study was to evaluate the immunogenicity and safety of a booster dose of MenACYW-TT 3–6 years after a priming vaccination with either MenACYW-TT or MCV4-CRM (Menveo, GSK) in adolescents and adults in the US, with and without co-administration of a MenB vaccine, and to evaluate the persistence of the immune response to MenACYW-TT and MCV4-CRM 3–6 years after vaccination.

## Methods

### Study design and participants

This was a phase IIIb, open-label, partially randomized, parallel-group, active-controlled, multicenter study to evaluate the immunogenicity and safety of a booster dose of MenACYW-TT when administered alone or co-administered with licensed MenB vaccines in adolescents and adults (WHO Universal Trial Number [UTN]: U1111-1217-2137; Clinicaltrials.gov, NCT04084769). The study was conducted between September 2019 and September 2020 at 29 centers in the United States and 1 center in Puerto Rico.

The study was approved by the appropriate independent ethics committees and institutional review boards before its initiation. The conduct of this study was consistent with standards established by the Declaration of Helsinki and compliant with the International Conference on Harmonization guidelines for Good Clinical Practice, including all local and/or national regulations, and directives.

Participants were eligible for inclusion if they were aged ≥13 to <26 years on the day of screening, and had participated in and completed any of two previous studies of MenACYW-TT (MenQuadfi®; Sanofi, Swiftwater, Pennsylvania) in the US (MET50 [NCT02199691] or MET43 [NCT02842853]),^21,25^ or had received a routine MCV4-CRM vaccine (Menveo®, GSK Vaccines, Sovicille, Italy) 3–6 years prior. Exclusion criteria included anyone who had received any vaccine in the 4 weeks preceding the study vaccine, except for influenza which could be received at least 2 weeks prior to the study, and had received any other meningococcal vaccination since the participation in the previous study of MenACYW-TT, had a known or suspected congenital/acquired immunodeficiency or had received immunosuppressive therapy or systemic corticosteroid therapy (for ≥2 consecutive weeks) within 6 or 3 months prior to the study, respectively, had a personal history of Guillain–Barré syndrome, or had received oral or injectable antibiotic therapy within 72 h prior to the first blood draw. Participants aged ≥13 to <18 years of age signed an assent form and their parent or guardian signed and dated the informed consent form (ICF) before any procedure or treatment associated with the trial performed. Those ≥18 years of age signed and dated the ICF themselves.

### Procedures

Participants who had previously received a priming dose of MenACYW-TT (MenACYW-TT primed) were randomized 2:1:1 to receive a booster dose of MenACYW-TT either alone (Group 1), or co-administered with a single dose of a licensed MenB vaccine, MenB-T (Trumenba®, Pfizer, Philadelphia; Group 3) or 4CMenB (Bexsero, GSK Vaccines, Sovicille, Italy; Group 4). Participants who had previously received a priming dose of MCV4-CRM were assigned to receive a booster dose of MenACYW-TT vaccine alone (Group 2). Only laboratory technicians were blinded to treatment assignment.

The booster dose of MenACYW-TT was administered intramuscularly (IM) into the deltoid muscle of the arm. Each MenACYW-TT dose was presented in 0.5 mL of saline solution containing 10 μg of each meningococcal capsular polysaccharide serogroups A, C, Y and W, and approximately 55 μg of tetanus toxoid protein carrier. MenB-T and 4CMenB were administered IM into the deltoid muscle of the opposite arm to that used for MenACYW-TT administration. Each MenB-T dose was presented in 0.5 mL containing factor H binding protein (fHBP) subfamily A, fHBP subfamily B, adsorbed on AIPO_4_. Each 4CMenB dose was presented as 0.5 mL containing recombinant *N. meningitidis* group B Neisseria Heparin Binding Antigen, group B Neisseria adhesin A protein, group B fHbp fusion protein and outer membrane vesicles from *N. meningitidis* group B strain NZ98/254 adsorbed on Al(OH)_3_. Second and third (if applicable) MenB doses were offered 30 and 180 days after the first MenB dose, respectively, in line with the US FDA-approved schedules for the respective MenB vaccines. These vaccinations were performed outside of study procedures and were not described in the study protocol.

### Immunogenicity

Blood samples for immunogenicity analyses were obtained prior to (Day 0) and 30 (+14) days after booster vaccination. A subset of the first 50 participants enrolled into groups 1 (MenACYW-TT primed, MenACYW-TT booster) and 2 (MCV4-CRM primed, MenACYW-TT booster) provided additional post-vaccination samples on Day 6 (±1 day).

Functional antibody titers against meningococcal serogroups A, C, W and Y were measured by a serum bactericidal antibodies assay using human complement (hSBA; Global Clinical Immunology, Sanofi, Swiftwater, PA), as described previously.^26^ The lower limit of quantification for the hSBA assay was 1:4. Vaccine seroresponse for serogroups A, C, Y and W was defined as hSBA pre-vaccination titer <1:8 for participants with a post-vaccination titer ≥1:16; or a ≥4-fold increase in titer post-vaccination for participants with a pre-vaccination titer ≥1:8. Vaccine seroprotection was defined as hSBA titers ≥1:8.

The primary immunogenicity endpoints of this study were vaccine seroresponse rates against meningococcal serogroups (A, C, W and Y) following a booster dose of MenACYW-TT in participants primed with MenACYW-TT 3–6 years previously, based on hSBA titers pre-booster and 30 days post booster. The secondary immunogenicity endpoints were hSBA seroresponse and seroprotection rates and geometric mean titers (GMTs) against serogroups A, C, W and Y in MenACYW-TT- or MCV4-CRM-primed participants, pre- (to evaluate persistence of immune response) and 30 days post-MenACYW-TT booster and, for a subset of participants, at 6 days post booster. In addition, vaccine hSBA seroresponse, seroprotection and GMTs to serogroups A, C, W and Y were determined following MenACYW-TT booster alone or co-administered with MenB-T or 4CMenB.

### Safety

Participants were observed for 30 min after vaccination to ensure their safety, during which, any unsolicited systemic adverse event (AE) was recorded. The occurrence of solicited injection site (pain, erythema and swelling) and systemic reactions (fever, headache, malaise and myalgia) were recorded up to 7 days after vaccination in the participant’s diary card provided to the parents/guardians. Parents or guardians were asked to inform the investigators of any potential serious adverse events (SAEs) immediately.

Unsolicited AEs were collected from Day 0 to Day 30 and medically attended AEs (MAAEs) and (SAEs) including AEs of special interest (AESIs) were recorded throughout the study (up to the 6-month [+14 days] follow-up phone call). Adverse events classified as AESIs were generalized seizures (febrile and non-febrile), Kawasaki disease, Guillain–Barré syndrome and idiopathic thrombocytopenia purpura.

Adverse events were assessed by the investigator for relatedness to the study vaccine and for intensity (grade 1 intensity [mild] to grade 3 [severe; interrupts usual daily activity]).

### Statistical analyses

A total of approximately 600 participants were planned to be enrolled. Three full analysis sets (FAS) were defined: FAS1 included participants who received at least one study vaccine booster dose and had a valid Day 6 serology result; FAS2 included participants who received at least one booster dose and had a valid Day 30 serology result; and FAS3 (for antibody persistence) included participants who had a valid Day 0 serology result. Two per protocol analysis sets (PPAS) were defined: PPAS1 for Day 6, and PPAS2 for Day 30 samples, each comprising participants from FAS1 or FAS2, respectively, who had no protocol deviations. Participant data in the FAS and PPAS were analyzed according to the groups that the participants were randomized to.

The safety analysis set (SafAS) was defined as all participants who received at least one dose of the study vaccine(s) and had safety data available. Safety was analyzed for participants in the SafAS according to the vaccine(s) they had received at Day 0.

Immunogenicity analyses were performed in the FAS2 and PPAS2 for Day 30 results and in the FAS1 and PPAS1 for Day 6 results; antibody persistence was assessed on the FAS3. Vaccine seroresponse sufficiency (primary objective) was demonstrated if the lower limit of the 1-sided 97.5% confidence interval (CI) calculated using the Clopper–Pearson method for the percentage of participants with hSBA vaccine seroresponse against serogroups A, C, W and Y, was greater than 75%. Seroresponse sufficiency was evaluated separately for Group 1 (MenACYW-TT primed, MenACYW-TT booster) and Group 2 (MCV4-CRM primed, MenACYW-TT booster). Data from participants of the MET50 and MET43 studies were used to evaluate antibody persistence and overall trends over 3–6 years post-priming with MenACYW-YY or MCV4-CRM. Antibody titers and corresponding 95% CIs were calculated on Log10 transformed data, assuming a normal distribution for the transformed data, with antilog transformations applied to provide GMTs and their 95% CIs. Participants who were previously primed with MCV4-CRM outside of prior studies were not included in the assessment of antibody persistence.

## Results

### Study participants

**Fig. 1:**
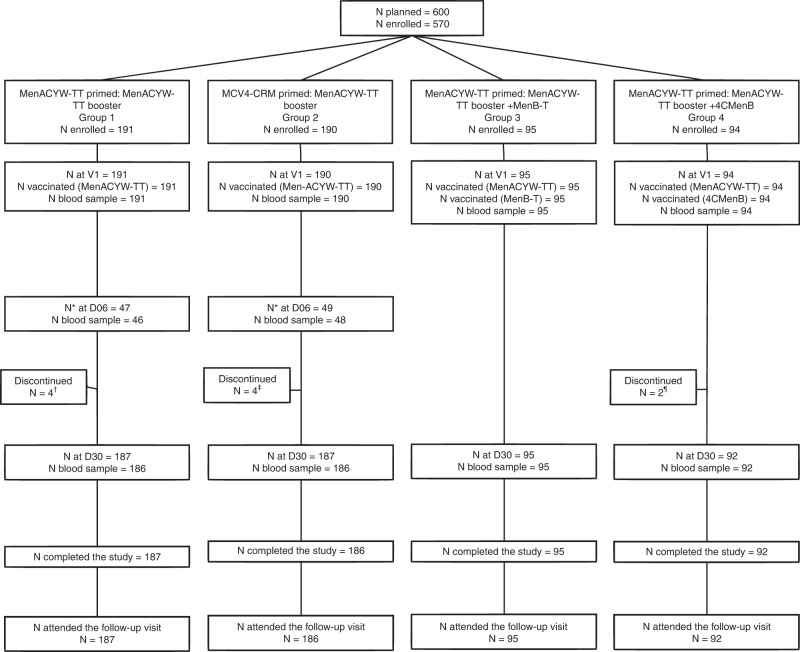
Participant disposition flow chart. *subset cohort† one participant was withdrawn by parent and Day 30 visit not conducted due to COVID-19, two participants were lost to follow up and Day 30 visit not conducted due to COVID-19, and one participant was enrolled in error (previously vaccinated with another meningococcal vaccine)‡ Day 30 visit was not conducted (due to COVID-19 for 3/4 participants)¶ Day 30 visit was not conducted for one participant due to parental concern about COVID-19, and one participant was enrolled in error (previously vaccinated with another meningococcal vaccine).

A total of 570 participants were enrolled in the study: 381 from the MET50 trial and 140 from the MET43 trial who had received either MenACYW-TT or MCV4-CRM, and 49 participants who received MCV4-CRM as part of routine immunization. Of these, 191 Group 1 (MenACYW-TT primed) participants and 190 Group 2 (MCV4-CRM primed) participants received MenACYW-TT booster alone, 95 MenACYW-TT primed participants received MenACYW-TT booster +MenB-T (Group 3) and 94 MenACYW-TT primed participants received the MenACYW-TT booster +4CMenB (Group 4) (Table 1 and Fig. 1). A total of 560 (98.2%) participants completed the trial (Fig. 1). The mean (SD) age of participants was 15.4 (1.4) years and was balanced across vaccine groups (Table 1). The majority of participants were white (87.2%), and the male:female ratio varied from 0.93 to 1.32 across the groups (Table 1).

**Table 1:** Baseline demographics (randomized and assigned groups).

	Group 1	Group 2	Group 3	Group 4
	(*N* = 191)	(*N* = 190)	(*N* = 95)	(*N* = 94)
Sex, *n* (%)				
Male	92 (48.2)	105 (55.3)	54 (56.8)	49 (52.1)
Female	99 (51.8)	85 (44.7)	41 (43.2)	45 (47.9)
Sex ratio, male:female	0.93	1.24	1.32	1.09
Age (years)				
Mean age, years (SD)	15.4 (1.6)	15.8 (1.4)	15.1 (1.1)	15.3 (1.3)
Min, max	13.0, 24.0	13.0, 23.0	13.0, 18.0	13.0, 22.0
Race, *n* (%)				
White	163 (85.3)	165 (86.8)	83 (87.4)	86 (91.5)
Asian	0	0	0	0
Black or African American	19 (9.9)	16 (8,4)	7 (7.4)	7 (7.4)
American Indian or Alaska Native	0	0	0	0
Native Hawaiian or Pacific Islander	1 (0.5)	0	0	1 (1.1)
Mixed	8 (4.2)	6 (3.2)	5 (5.3)	0
Not reported	0	2 (1.1)	0	0
Unknown	0	1 (0.5)	0	0

Group 1, MenACYW-TT primed: MenACYW-TT booster; Group 2, MCV4-CRM primed: MenACYW-TT booster; Group 3, MenACYW-TT booster +MenB-T; Group 4, MenACYW-TT booster +4CMenB.

*n* number of subjects with baseline demographic, *N* number of subjects in all randomized or assigned groups, *SD* standard deviation.

### Immunogenicity

#### Immunogenicity of the MenACYW-TT booster dose

The primary endpoint of the sufficiency of the vaccine seroresponse to serogroups A, C, W and Y at Day 30 following a booster dose of MenACYW-TT alone was achieved in Group 1 and 2 participants (Table 2). At 30 days after a booster dose of MenACYW-TT, seroresponse was seen in >93% of participants across serogroups, regardless of the priming vaccination (Table 2).

**Table 2:** Sufficiency^a^ of the proportion of participants with an hSBA vaccine seroresponse^b^ to serogroups A, C, W and Y at Day 30 following MenACYW-TT booster in Group 1 and Group 2 (PPAS2).

	Group 1 (*N* = 174)				Group 2 (*N* = 176)			
Serogroup	*n*/*M*	% (95% CI)	Lower limit of 1-sided 97.5% CI	Sufficiency^a^	*n*/*M*	% (95% CI)	Lower limit of 1-sided 97.5% CI	Sufficiency^a^
A	165/174	94.8 (90.4, 97.6)	90.4	Yes	164/176	93.2 (88.4, 96.4)	88.4	Yes
C	169/174	97.1 (93.4, 99.1)	93.4	Yes	174/176	98.9 (96.0, 99.9)	96.0	Yes
W	170/174	97.7 (94.2, 99.4)	94.2	Yes	174/176	98.9 (96.0, 99.9)	96.0	Yes
Y	172/174	98.9 (95.9, 99.9)	95.9	Yes	176/176	100 (97.9, 100)	97.9	Yes

Group 1, MenACYW-TT primed: MenACYW-TT booster; Group 2, MCV4-CRM primed: MenACYW-TT booster.

*CI* confidence interval, *n* number of participants with seroresponse, *N* number of participants in PPAS2, *M* number of participants with valid serology results.

^a^hSBA vaccine seroresponse sufficiency was demonstrated if the lower limit of the 1-sided 97.5% CI > 75%.

^b^For a participant with a pre-vaccination titer <1:8, the post-vaccination titer must be ≥1:16; For a participant with a pre-vaccination titer ≥1:8, the post-vaccination titer must be at least 4-fold greater than the pre-vaccination titer.

On Day 30 after MenACYW-TT booster vaccination, nearly all participants demonstrated hSBA vaccine seroprotection against each serogroup in Groups 1 and 2 (serogroup A, 99.4% [95% CI 96.8, 100] and 99.4% [95% CI 96.9, 100]; serogroup C, 100.0% [95% CI 97.9, 100] and 100% [95% CI 07.9, 200]; serogroup W, 100% [95% CI 97.9, 100] and 100% [95% CI 97.9, 100]; and serogroup Y, 100% [95% 97.9, 100] and 100% [95% 97.9, 100], respectively). Day 30 GMTs were also comparable between Group 1 and Group 2 for serogroups A, W and Y, with higher GMTs for serogroup C in Group 1 (Table 3).

**Table 3:** GMTs (hSBA) 30 days after a MenACYW-TT booster in MenACYW-TT-primed and MCV4-CRM primed participants (PPAS2).

	Group 1 (*N* = 174)			Group 2 (*N* = 176)			Group 1/Group 2	
Serogroup	*M*	GMT	(95% CI)	*M*	GMT	(95% CI)	GMTR	(95% CI)
A	174	502	(388, 649)	176	399	(318, 502)	1.26	(0.89, 1.77)
C	174	3708	(3146, 4369)	176	2533	(2076, 3091)	1.46	(1.13, 1.89)
W	174	2290	(1934, 2711)	176	2574	(2178, 3041)	0.89	(0.70, 1.13)
Y	174	2308	(1925, 2767)	176	3036	(2547, 3620)	0.76	(0.59, 0.98)

Group 1, MenACYW-TT primed: MenACYW-TT booster; Group 2, MCV4-CRM primed: MenACYW-TT booster.

*CI* confidence interval, *GMT* geometric mean titer, *GMTR* geometric mean titer ratio, *M* number of participants with valid serology results, *N* number of participants in PPAS2.

**Fig. 2:**
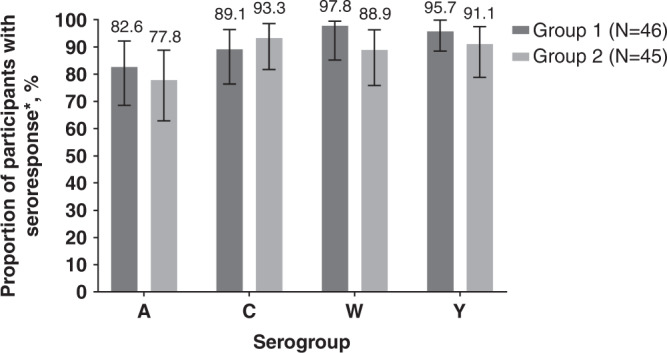
Proportion of participants with hSBA vaccine seroresponse at Day 6 post-MenACYW-TT booster. Proportion of participants with hSBA vaccine seroresponse at Day 6 post-MenACYW-TT booster in MenACYW-TT primed (Group 1) and MCV4-CRM primed (Group 2) participants (PPAS1). Errors bars indicate 95% confidence intervals. Group 1, MenACYW-TT primed: MenACYW-TT booster; Group 2, MCV4-CRM primed: MenACYW-TT booster. *For a participant with a pre-vaccination titer <1:8, the post-vaccination titer must be ≥1:16; for a participant with a pre-vaccination titer ≥1:8, the post-vaccination titer must be at least 4-fold greater than the pre-vaccination titer

The proportion of participants achieving a vaccine seroresponse 6 days after a booster dose of MenACYW-TT alone was high for each serogroup, regardless of the priming vaccine, ranging from 82.6% [95% CI 68.6, 92.2] to 97.8% [95% CI 88.5, 99.9] in Group 1 and from 77.8% [95% CI 62.9, 88.8] to 93.3% [95% CI 81.7, 98.6] in Group 2 (Fig. 2).

Most participants in both groups demonstrated hSBA seroprotection by Day 6 post booster (Serogroup A, 91.3% [95% CI 79.2, 97.6] and 95.6% [95% CI 84.9, 99.5]; Serogroup C, 100% [95% CI 92.3, 100] and 97.8% [95% CI 88.2, 99.9]; Serogroup W, 100% [95% CI 92.3, 100] and 100% [95% CI 92.1, 100]; and Serogroup Y, 97.8% [95% CI 88.5, 99.9] and 100.0% [95% CI 92.1, 100], in Group 1 and Group 2, respectively). The Day 6 GMTs were also comparable in Group 1 and Group 2 for serogroups A and Y, and were higher for serogroups C and W in Group 1 (Supplementary Table S1).

#### Persistence of the immune response to MenACYW-TT and MCV4-CRM priming vaccinations administered 3–6 years earlier

**Fig. 3:**
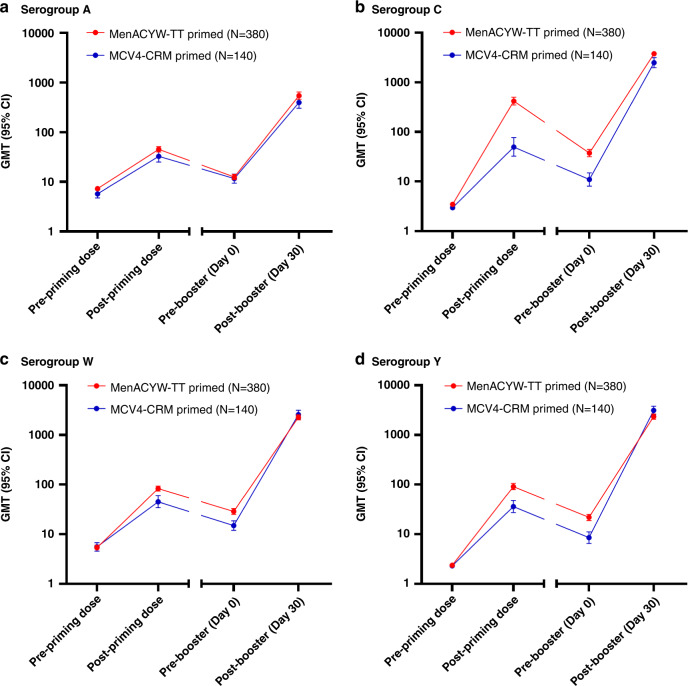
Persistence of hSBA GMTs following priming vaccination with MenACYW-TT or MCV4-CRM. Persistence of hSBA GMTs to serogroup A (**a**), serogroup C (**b**), serogroup W (**c**) and serogroup Y (**d**) following priming vaccination with MenACYW-TT or MCV4-CRM (FAS3). CI, confidence interval; GMT, geometric mean titer; N, total subjects; hSBA human complement serum bactericidal antibody assay. MenACYW-TT primed (red line), Group 1, 3 and 4 participants who were MenACYW-TT primed in the previous studies, MET50 or MET43; MCV4-CRM primed (blue line), Group 2 participants who were MCV4-CRM primed in the previous study, MET50.

Pre- and post-primary vaccination data from the initial MET50 and MET43 studies showed increases in GMTs for all four serogroups. GMTs then declined over the 3–6 years following this priming vaccination, but remained higher than pre-vaccination levels for those participants with the available data from previous trials (Fig. 3). Three to six years following the priming vaccination the GMTs for serogroups C, W and Y were higher in those with MenACYW-TT priming vaccination (Groups 1, 3, and 4) than MCV4-CRM priming vaccination (Group 2), and comparable for serogroup A (Fig. 3). Similarly, at Day 0 before booster, more than 50% of participants in each vaccine group demonstrated hSBA vaccine seroprotection, with over 85% of those primed with MenACYW-TT demonstrating seroprotection to serogroups C and W 3–6 years after the priming vaccination (Supplementary Table S2).

#### Co-administration of MenACYW-TT vaccine with MenB vaccines

**Fig. 4:**
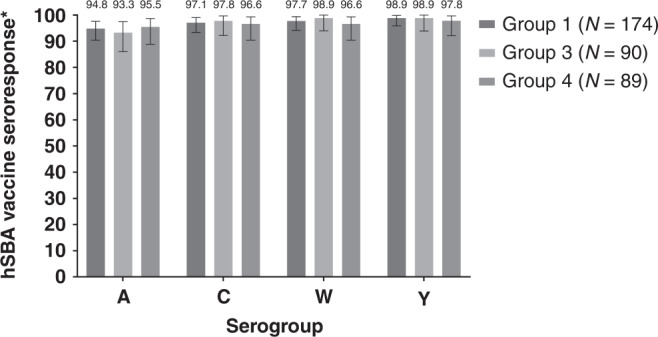
Percentage of subjects achieving hSBA vaccine seroresponse at Day 30 post-booster with MenACYW-TT booster alone, or co-administered with MenB-T or 4CMenB. Errors bars indicate 95% confidence intervals. hSBA, human complement serum bactericidal antibody assay Group 1, MenACYW-TT primed: MenACYW-TT booster; Group 3, MenACYW-TT primed: MenACYW-TT booster +MenB-T; Group 4, MenACYW-TT primed: MenACYW-TT booster +4CMenB *For a participant with a pre-vaccination titer <1:8, the post-vaccination titer must be ≥1:16; for a participant with a pre-vaccination titer ≥1:8, the post-vaccination titer must be at least 4-fold greater than the pre-vaccination titer

In MenACYW-TT primed participants, seroresponse at Day 30 was comparable between Groups 1, 3 and 4 (Fig. 4). Seroprotection to each of the serogroups was also comparable across these groups (Supplementary Table S3), as were GMTs (Table 4).

**Table 4:** GMTs (hSBA) to each of the serogroups at Day 0 and Day 30 in MenACYW-TT primed participants who received MenACYW-TT booster alone or co-administered with a MenB vaccine (PPAS2).

		Group 1 (*N* = 174)			Group 3 (*N* = 90)			Group 4 (*N* = 89)			Group 1/Group 3		Group 1/Group 4	
Serogroup	Time point	*M*	GMT	(95% CI)	*M*	GMT	(95% CI)	*M*	GMT	(95% CI)	GMTR	(95% CI)	GMTR	(95% CI)
A	D 0	174	11.7	(9.89, 13.8)	90	12.5	(9.78, 16.0)	88	12.3	(9.37, 16.2)	0.934	(0.699, 1.25)	0.947	(0.699, 1.28)
	D 30	174	502	(388, 649)	90	593	(426, 825)	89	667	(477, 933)	0.847	(0.552, 1.30)	0.752	(0.489, 1.16)
C	D 0	174	36.6	(28.8, 46.7)	90	37.6	(26.6, 53.3)	89	42.4	(29.4, 61.0)	0.974	(0.642, 1.48)	0.865	(0.566, 1.32)
	D 30	174	3708	(3146, 4369)	90	4741	(3882, 5791)	88	3472	(2667, 4518)	0.782	(0.597, 1.02)	1.07	(0.795, 1.44)
W	D 0	174	27.0	(22.0, 33.1)	90	28.3	(22.0, 36.4)	88	30.0	(22.5, 40.1)	0.953	(0.681, 1.33)	0.897	(0.631, 1.28)
	D 30	174	2290	(1934, 2711)	90	2702	(2134, 3422)	89	2064	(1601, 2662)	0.847	(0.635, 1.13)	1.11	(0.824, 1.49)
Y	D 0	174	20.5	(16.6, 25.2)	89	25.5	(19.4, 33.6)	89	21.0	(15.4, 28.7)	0.802	(0.565, 1.14)	0.975	(0.677, 1.40)
	D 30	174	2308	(1925, 2767)	90	2600	(2042, 3311)	89	2469	(1881, 3241)	0.888	(0.654, 1.20)	0.935	(0.680, 1.28)

Group 1, MenACYW-TT primed: MenACYW-TT booster; Group 3, MenACYW-TT booster +MenB-T; Group 4, MenACYW-TT booster +4CMenB.

*CI* confidence interval, *GMTR* geometric mean titer ratio, *M* number of participants with valid serology results for the particular subgroup, *N* number of participants in PPAS2.

#### Safety

No participants in Groups 1, 3 or 4 reported an immediate unsolicited event. One participant in Group 2 (0.5% [1/184]) experienced an immediate unsolicited AE of Grade 2 presyncope, related to the study vaccination. Solicited reactions were reported at similar frequencies between Group 1 and Group 2 participants (≥1 solicited injection site reaction, 42.5% [79/186] and 35.9% [66/184], respectively; ≥1 solicited systemic reaction, 54.3% [101/186] and 54.3% [100/184], respectively. Higher frequencies of solicited reactions were reported overall for the two MenB co-administration booster groups, Groups 3 and 4 (≥1 solicited injection site reaction, 83.7% [77/92] and 92.4% [85/92]; ≥1 solicited systemic reaction, 75.0% [69/92] in each group) (Table 5). Injection site pain was the most frequently reported solicited injection site reaction in each group, occurring at the highest frequency in Groups 3 and 4. The most frequent solicited systemic reactions were headache, malaise and myalgia in Group 1 and Group 2, and myalgia in Groups 3 and 4; myalgia was reported more frequently in the MenB co-administration booster groups than in the other groups (Supplementary Table S4). Solicited injection site reactions and solicited systemic reactions were mostly of mild (grade 1) intensity (Supplementary Table S5)

**Table 5:** Safety overview.

	Group 1 (*N* = 186)			Group 2 (*N* = 184)			Group 3 (*N* = 93)			Group 4 (*N* = 92)		
Subjects experiencing at least one:	*n*/*N*	%	(95% CI)	*n*/*N*	%	(95% CI)	*n*/*N*	%	(95% CI)	*n*/*N*	%	(95% CI)
Within 30 min after vaccination												
Immediate unsolicited AE	0/186	0	(0, 2.0)	1/184	0.5	(0. 3.0)	0/93	0	(0, 3.9)	0/92	0	(0, 3.9)
Immediate unsolicited AR	0/186	0	(0, 2.0)	1/184	0.5	(0. 3.0)	0/93	0	(0, 3.9)	0/92	0	(0, 3.9)
Within 7 days after vaccination												
Solicited reaction	126/186	67.7	(60.5, 74.4)	11/184	59.8	(52.3, 66.9)	82/92	89.1	(80.9, 94.7)	88/92	95.7	(89.2, 98.8)
Solicited injection site reaction	79/186	42.5	(35.3, 49.9)	66/184	35.9	(28.9, 43.3)	77/92	83.7	(74.5, 90.6)	85/92	92.4	(84.9, 96.9)
MenACYW-TT	79/186	42.5	(35.3, 49.9)	66/184	35.9	(28.9, 43.3)	47/92	51.1	(40.4, 61.7)	52/92	56.85	(45.8, 66.8)
MenB-T	NA	NA	NA	NA	NA	NA	69/92	75.0	(64.9, 83.4)	NA	NA	NA
4CMenB	NA	NA	NA	NA	NA	NA	NA	NA	NA	71/92	77.2	(67.2, 85.3)
Solicited systemic reactions	101/186	54.3	(46.9, 61.6)	100/184	54.3	(46.9, 61.7)	69/92	75.0	(64.9, 83.4)	69/92	75.0	(64.9, 83.4)
Within 30 days after vaccination												
Unsolicited AE	43/186	23.1	(17.3, 29.8)	49/184	26.6	(20.4, 33.6)	23/93	24.7	(16.4, 34.8)	24/92	26.1	(17.5, 36.3)
Unsolicited AR	5/186	2.7	(0.9, 6.2)	7/184	3.8	(1.5, 7.7)	2/93	2.2	(0.3, 7.6)	3/92	3.3	(0.7, 9.2)
Unsolicited non-serious AE	43/186	23.1	(17.3, 29.8)	49/184	26.6	(20.4, 33.6)	23/93	24.7	(16.4, 34.8)	24/92	26.1	(17.5, 36.3)
Unsolicited non-serious AR	5/186	2.7	(0.9, 6.2)	7/184	3.8	(1.5, 7.7)	2/93	2.2	(0.3, 7.6)	3/92	3.3	(0.7. 9.2)
Unsolicited non-serious injection site AR	4/186	2.2	(0.6, 5.4)	2/184	1.1	(0.1, 3.9)	0/93	0	(0, 3.9)	0/92	0	(0, 3.9)
MenACYW-TT	4/186	2.2	(0.6, 5.4)	2/184	1.1	(0.1, 3.9)	0/93	0	(0, 3.9)	0/92	0	(0, 3.9)
MenB-T	NA	NA	NA	NA	NA	NA	0/93	0	(0, 3.9)	NA	NA	NA
4CMenB	NA	NA	NA	NA	NA	NA	NA	NA	NA	0/92	0	(0, 3.9)
Unsolicited non-serious systemic AE	39/186	21.0	(15.4, 27.5)	47/184	25.5	(19.4, 32.5)	23/93	24.7	(16.4, 34.8)	24/92	26.1	(17.5, 36.3)
Unsolicited non-serious systemic AR	1/186	0.5	(0, 3.0)	5/184	2.7	(0.9, 6.2)	2/93	2.2	(0.3, 7.6)	3/92	3.3	(0, 3.9)
AE leading to study discontinuation	0/186	0	(0, 2.0)	0/184	0	(0, 2.0)	0/93	0	(0, 3.9)	0/92	0	(0, 3.9)
AR leading to study discontinuation	0/186	0	(0, 2.0)	0/184	0	(0, 2.0)	0/93	0	(0, 3.9)	0/92	0	(0, 3.9)
SAE	0/186	0	(0, 2.0)	0/184	0	(0, 2.0)	0/93	0	(0, 3.9)	0/92	0	(0, 3.9)
Related SAE	0/186	0	(0, 2.0)	0/184	0	(0, 2.0)	0/93	0	(0, 3.9)	0/92	0	(0, 3.9)
Death	0/186	0	(0, 2.0)	0/184	0	(0, 2.0)	0/93	0	(0, 3.9)	0/92	0	(0, 3.9)
AESI	0/186	0	(0, 2.0)	0/184	0	(0, 2.0)	0/93	0	(0, 3.9)	0/92	0	(0, 3.9)
MAAE	9/186	4.8	(2.2, 9.0)	13/184	7.1	(3.8, 11.8)	7/93	7.5	(3.1, 14.9)	9/92	9.8	(4.6, 17.8)
During the study												
SAE	2/186	1.1	(0.1, 3.8)	2/184	1.1	(0.1, 3.9)	2/93	2.2	(0.3, 7.6)	0/92	0	(0, 3.9)
Related SAE	0/186	0	(0, 2.0)	0/184	0	(0, 2.0)	0/93	0	(0, 3.9)	0/92	0	(0, 3.9)
Death	0/186	0	(0, 2.0)	0/184	0	(0, 2.0)	0/93	0	(0, 3.9)	0/92	0	(0, 3.9)
AESI	0/186	0	(0, 2.0)	0/184	0	(0, 2.0)	0/93	0	(0, 3.9)	0/92	0	(0, 3.9)

Group 1, MenACYW-TT primed: MenACYW-TT booster; Group 2, MCV4-CRM primed: MenACYW-TT booster; Group 3, MenACYW-TT booster +MenB-T; Group 4, MenACYW-TT booster +4CMen.

*AE* adverse event, *AESI* adverse event of special interest, *AR* adverse reaction, *CI* confidence interval, *MAAE* medically attended adverse event, *n* number of subjects with the outcome listed, *N* number of subjects in Safety Analysis Set, *SAE* serious adverse event.

No participants experienced a serious AE (SAE) within 30 days of vaccination, and no participants experienced AEs or adverse reaction (AR) which led to study discontinuation. During the study, six SAEs were reported: two cases of Grade 3 appendicitis (one each in Group 1 and Group 2), two cases of Grade 3 suicidal ideation (one each in Group 1 and Group 2), one case of Grade 3 major depression and suicidal ideation (Group 3) and one case of Grade 3 accidental overdose (Group 3). All occurred between Day 31 and the 6-month follow-up contact and were not considered related either by the investigator or by the sponsor. No deaths or AEs of special interest were reported.

## Discussion

This phase IIIb, open-label, partially randomized, parallel-group, multicenter trial demonstrated that a booster dose of MenACYW-TT in adolescents and adults who had been vaccinated 3–6 years earlier with a single dose of MenACYW-TT or MCV4-CRM was immunogenic and well tolerated, with in the sufficiency of seroresponse demonstrated at Day 30 post booster. We observed a robust response to a booster dose of MenACYW-TT, with over 93% seroresponse for all serogroups in adolescents and adults. The seroresponse rate was greater than 75% for all serogroups in both Groups 1 and 2 by Day 6, suggesting a quick onset of the immune response, regardless of the priming vaccine (MenACYW-TT or MCV4-CRM). This observation supports previous studies of a booster dose of MenACYW-TT which have shown similar results,^23,24^ and is indicative of immune memory in these participants. Such a rapid anamnestic response is important to be able to rapidly boost protection against infection among groups of at-risk individuals whose protective antibody levels may have waned over time and thus may otherwise be at risk of a potential outbreak. Vaccination with MenACYW-TT as a booster in adolescents and adults vaccinated 3–6 years earlier with MenACYW-TT or MCV4-CRM was found to be well tolerated with no safety concerns identified. There were no SAEs within 30 days of the booster, or discontinuations due to AEs.

When examining the persistence of the immune response, 3–6 years after the priming vaccination with either MenACYW-TT or MCV4-CRM, prior to the booster vaccination, seroprotection ranged from 52% to 91% across the serogroups and vaccine groups. GMTs at Day 0, pre-booster, were higher than those prior to the priming vaccine in participants from previous studies,^20,25^ suggesting the persistence of the immune response in these participants. GMTs were also higher pre-booster in those primed with MenACYW-TT compared to those primed with MCV4-CRM for serogroups C, W and Y. These results demonstrate for the first time persistence of the immune response in adolescents and young adults and are in line with previous studies of a booster dose of MenACYW-TT findings showing persistence of the immune response in a younger population.^24^

Based on the GMTRs at Day 30 post booster dose for Groups 1 (MenACYW-TT primed) and 2 (MCV4-CRM-primed), possible evidence of a statistically significant difference for serogroups C and Y (considering 95% CIs do not cross 1) was observed; however, it must be noted that a formal statistical hypothesis to evaluate such a difference was not a part of the planned analysis and is a post-hoc observation. The co-administration of the MenACYW-TT booster with a MenB vaccine did not affect the immunogenicity of serogroups A, C, Y and W, compared to the administration of the MenACYW-TT booster alone.

Solicited AEs were mostly of mild intensity but showed a numerical increase, particularly in malaise and myalgia, likely influenced by the known reactogenic profile of MenB vaccines.^27–32^ Co-administration of these vaccines will be important in a number of countries globally, including the US, to facilitate the provision of broader protection against IMD particularly in higher-risk groups such as adolescents.

While this study assessed the co-administration of MenACYW-TT and MenB vaccines, the immunogenicity of MenB vaccines when co-administered with MenACYW-TT was not assessed in this study due to non-availability of relevant assays. It will be important to address this in future studies. In addition, we did not have pre- or post-priming vaccine data for the 49 participants who had not participated in the previous MET50 or MET43 clinical studies and as such these participants were not included in the analysis of antibody persistence. This study may also have benefitted from the inclusion of an additional group of participants as a control to assess the response of a single dose of MenACYW-TT in vaccine-naïve participants; however, studies showing robust immunogenicity of a single dose of MenACYW-TT in this age group have been published previously.^20,25^

This study offers key data about the immunogenicity and safety of MenACYW-TT when administered as a booster alone or co-administered with a MenB vaccine, in participants primed with MenACYW-TT or MCV4-CRM. A booster dose of MenACYW-TT produced a comparable and robust response to all serogroups in adolescents and adults who had received a priming dose of either MenACYW-TT or MCV4-CRM 3–6 years earlier. In addition, no interference of the immune response to MenACYW-TT was observed when MenACYW-TT was co-administered with MenB-T or 4CMenB vaccines.

## Supplementary information

Supplementary Materials

## Data Availability

Qualified researchers may request access to patient-level data and related documents [including, e.g., the clinical study report, study protocol with any amendments, blank case report form, statistical analysis plan, and dataset specifications]. Patient-level data will be anonymized, and study documents will be redacted to protect the privacy of trial participants. Further details on Sanofi’s data-sharing criteria, eligible studies, and process for requesting access can be found at https://vivli.org/.
