# Correction: Quadrivalent meningococcal tetanus toxoid-conjugate booster vaccination in adolescents and adults: phase III randomized study

**DOI:** 10.1038/s41390-023-02835-4

**Published:** 2023-10-18

**Authors:** Betzana Zambrano, James Peterson, Carmen Deseda, Katie Julien, Craig A. Spiegel, Clifford Seyler, Michael Simon, Robert Hoki, Marc Anderson, Brad Brabec, Germán Áñez, Jiayuan Shi, Judy Pan, Audrey Hagenbach, Dalia Von Barbier, Kucku Varghese, Emilia Jordanov, Mandeep Singh Dhingra

**Affiliations:** 1 Global Clinical Development Strategy, Sanofi, Montevideo, Uruguay; 2 J. Lewis Research, Salt Lake City, UT USA; 3 Caribbean Travel Medicine Clinic, San Juan, Puerto Rico; 4 J. Lewis Research Inc, South Jordan, UT USA; 5 Craig A. Spiegel, MD Bridgeton, Bridgeton, MO USA; 6 Pediatric Clinical Trials Tullahoma, Tullahoma, TN USA; 7 Michael W. Simon, MD, PSC, Lexington, KY USA; 8 Wee Care Pediatrics, Layton, UT USA; 9 Tanner Clinic, Layton, UT USA; 10 Midwest Children’s Health Research Institute, Lincoln, NE USA; 11 Global Biostatistical Sciences, Sanofi, Swiftwater, PA USA; 12 Sanofi, Marcy L’Étoile, France; 13 Sanofi, Swiftwater, PA USA; 14 Present Address: Global Clinical Development Strategy, Sanofi, Swiftwater, PA USA; 15 Present Address: Global Clinical Immunology, Sanofi, Swiftwater, PA USA

Correction to: *Pediatric Research* 10.1038/s41390-023-02478-5, published online 10 March 2023

In the sentence beginning ‘On Day 30 after MenACYW-TT booster vaccination…’ in this article, the value ‘100% [95% CI 07.9, 200]’ should have read ‘100% [95% CI 97.9, 100]’.

The original article has been corrected.

