# Supplementary material for: Quadrivalent meningococcal tetanus toxoid-conjugate booster vaccination in adolescents and adults: phase III randomized study
Source: Pediatr Res. 2023 Mar 10;94(3):1035–43. doi: 10.1038/s41390-023-02478-5 (PMC10000353; doi:10.1038/s41390-023-02478-5)
Supplement: Supplementary file 1 — Supplementary Materials [file 41390_2023_2478_MOESM1_ESM.pdf]

Supplementary Materials

Table S1: GMTs (hSBA) 6 days after a MenACYW-TT booster in Group 1 and Group 2 participants (PPAS1)

	Group 1 (N=46)			Group 2 (N=45)			Group 1/Group 2	
**Serogroup**	**M**	**GMT**	**(95% CI)**	**M**	**GMT**	**(95% CI)**	**GMTR**	**(95% CI)**
**A**	46	289	(133, 625)	45	161	(93, 280)	1.79	(0.70, 4.58)
**C**	46	3799	(2504, 5763)	45	919	(500, 1690)	4.13	(2.00, 8.53)
**W**	46	1928	(1187, 3131)	45	708	(463, 1082)	2.73	(1.44, 5.15)
**Y**	46	1658	(973, 2826)	45	800	(467, 1371)	2.07	(0.98, 4.38)

CI, confidence interval; GMT, geometric mean titer; GMTR, geometric mean titer ratio; M, number of participants with valid serology results; N, number of participants in PPAS1.

Group 1, MenACYW-TT primed: MenACYW-TT booster; Group 2; MCV4-CRM primed: MenACYW-TT booster

Table S2: Proportion of participants with seroprotection (hSBA titers ≥1:8) at Day 0 (Pre – booster vaccination FAS3)

	Pooled Groups 1, 3, 4 (N=380)			Group 2 (N=140)			Difference, % (95% CI)
**Serogroup**	**n/M**	**%**	**(95% CI)**	**n/M**	**%**	**(95% CI)**	
**A**	276/379	72.8	(68.0, 77.2)	100/140	71.4	(63.2, 78.7)	1.39 (-6.89, 10.43)
**C**	328/380	86.3	(82.4, 89.6)	69/140	49.3	(40.7, 57.9)	37.03 (27.99, 45.75)
**W**	337/379	88.9	(85.3, 91.9)	107/140	76.4	(68.5, 83.2)	12.49 (5.28, 20.65)
**Y**	310/379	81.8	(77.5, 85.5)	73/140	52.1	(43.5, 60.7)	29.65 (20..52, 38.61)

M, number of participants with valid serology results for the particular serogroup; n, number of participants meeting the endpoint; N, number of participants in FAS3.

The MenACYW-TT primed group comprises all participants who were MenACYW-TT primed in MET50 or MET43 (Group 1, MenACYW-TT primed: MenACYW-TT; Group 3, MenACYW-TT + MenB-T; and Group 4, MenACYW-TT + 4CMenB). The MCV4-CRM group comprises all participants who were MCV4-CRM primed in MET50 (Group 2).

Table S3: Seroprotection (hSBA titers ≥1:8) to each of the serogroups at Day 0 and Day 30 in MenACYW-TT primed participants who received MenACYW-TT booster alone or co-administered with a MenB vaccine (PPAS2)

		Group 1 (N=174)			Group 3 (N=90)			Group 4 (N=89)			Group 1- Group 3		Group 1- Group 4	
Serogroup	Time point	n/M	%	(95% CI)*	n/M	%	(95% CI)*	n/M	%	(95% CI)*	Difference (%)	(95% CI)**	Difference (%)	(95% CI)**
A	D 0	124/174	71.3	(63.9, 77.9)	66/90	73.3	(63.0, 82.1)	63/88	71.6	(61.0, 80.7)	-2.07	(-12.8, 9.66)	-0.33	(-11.31, 11.59)
	D 30	173/174	99.4	(96.8, 100)	89/90	98.9	(94.0, 100)	89/89	100	(95.9, 100)	0.54	(-2.23, 5.48)	-0.57	(-3.18, 3.59)
C	D 0	153/174	87.9	(82.1, 92.4)	78/90	86.7	(77.9, 92.9)	77/89	86.5	(77.6, 92.8)	1.26	(-6.67, 10.71)	1.41	(-6.56, 10.93)
	D 30	174/174	100	(97.9, 100)	90/90	100	(96.0, 100)	88/88	100	(95.9, 100)	0.0	(-2.16, 4.09)	0.00	(-2.16, 4.18)
W	D 0	150/174	86.2	(80.2, 91.0)	82/90	91.1	(83.2, 96.1)	80/88	90.9	(82.9, 96.0)	-10.0	(12.22, 3.92)	-4.70	(-12.08, 4.26)
	D 30	174/174	100	(97.9, 100)	90/90	100	(96.0, 100)	89/89	100	(95.9, 100)	0.0	(-2.16, 4.09)	0.00	(-2.16, 4.14)
Y	D 0	139/174	79.9	(73.2, 85.6)	80/89	89.9	(81.7, 95.3)	72/89	80.9	(71.2, 88.5)	-4.9	(-18.08, -0.42)	-1.01	(-10.48, 9.75)
	D 30	174/174	100	(97.9, 100)	90/90	100	(96.0, 100)	89/89	100	(95.9, 100)	0.0	(-2.16, 4.09)	0.00	(-2.16, 4.14)

CI, confidence interval; M, number of participants with valid serology results for the serogroup; n, number of participants with titers that meet the hSBA vaccine seroprotection (hSBA titer ⩾1:8) criteria; N, number of participants in PPAS2.

Group 1, MenACYW-TT primed: MenACYW-TT booster; Group 3, MenACYW-TT booster +MenB-T; Group 4, MenACYW-TT booster +4CMenB

*95% CI of the single proportion calculated from the exact binomial method; **95% CI of the difference calculated from the Wilson Score method without continuity correction

Table S4: Solicited injection site reactions after vaccine injection, by maximum intensity during the solicited period (Safety Analysis Set)

		Group 1 (N=186)			Group 2 (N=184)			Group 3 (N=93)			Group 4 (N=92)		
**Subjects experiencing at least one:**	**Maximum Intensity**	**n/M**	**%**	**(95% CI)**	**n/M**	**%**	**(95% CI)**	**n/M**	**%**	**(95% CI)**	**n/M**	**%**	**(95% CI)**
**MenACYW**													
Injection Site Pain	Any	71/186	38.2	(31.2, 45.6)	62/184	33.7	(26.9, 41.0)	45/92	48.9	(38.3, 59.6)	52/92	56.5	(45.8, 66.8)
	Grade 1	62/186	33.3	(26.6, 40.6)	51/184	27.7	(21.4, 34.8)	29/92	31.5	(22.2, 42.0)	33/92	35.9	(26.1, 46.5)
	Grade 2	8/186	4.3	(1.9, 8.3)	9/184	4.9	(2.3, 9.1)	11/92	12.0	(6.1, 20.4)	19/92	20.7	(12.9, 30.4)
	Grade 3	1/186	0.5	(0, 3.0)	2/184	1.1	(0.1, 3.9)	5/92	5.4	(1.8, 12.2)	0/92	0	(0, 3.9)
Injection Site Erythema	Any	12/186	6.5	(3.4, 11.0)	10/184	5.4	(2.6, 9.8)	1/92	1.1	(0, 5.9)	6/92	6.5	(2.4, 13.7)
	Grade 1	8/186	4.3	(1.9, 8.3)	9/184	4.9	(2.3, 9.1)	1/92	1.1	(0, 5.9)	2/92	2.2	(0.3, 7.6)
	Grade 2	3/186	1.6	(0.3, 4.6)	1/184	0.5	(0, 3.0)	0/92	0	(0, 3.9)	3/92	3.3	(0.7, 9.2)
	Grade 3	1/186	0.5	(0, 3.0)	0/184	0	(0, 2.0)	0/92	0	(0, 3.9)	1/92	1.1	(0, 5.9)
Injection Site Swelling	Any	10/186	5.4	(2.6, 9.7)	3/184	1.6	(0.3, 4.7)	2/92	2.2	(0.3, 7.6)	5/92	5.4	(1.8, 12.2)
	Grade 1	5/186	2.7	(0.9, 6.2)	1/184	0.5	(0, 3.0)	1/92	1.1	(0, 5.9)	3/92	3.3	(0.7, 9.2)
	Grade 2	5/186	2.7	(0.9, 6.2)	2/184	1.1	(0.1, 3.9)	1/92	1.1	(0, 5.9)	1/92	1.1	(0, 5.9)
	Grade 3	0/186	0	(0, 2.0)	0/184	0	(0, 2.0)	0/92	0	(0, 3.9)	1/92	1.1	(0, 5.9)
MenB-T													
Injection Site Pain	Any	NA	NA	NA	NA	NA	NA	69/92	75.0	(64.9, 83.4)	NA	NA	NA
	Grade 1	NA	NA	NA	NA	NA	NA	32/92	34.8	(25.1, 45.4)	NA	NA	NA
	Grade 2	NA	NA	NA	NA	NA	NA	29/92	31.5	(22.2, 42.0)	NA	NA	NA
	Grade 3	NA	NA	NA	NA	NA	NA	8/92	8.7	(3.8, 16.4)	NA	NA	NA
Injection Site Erythema	Any	NA	NA	NA	NA	NA	NA	14/92	15.2	(8.6, 24.2)	NA	NA	NA
	Grade 1	NA	NA	NA	NA	NA	NA	6/92	6.5	(2.4, 13.7)	NA	NA	NA
	Grade 2	NA	NA	NA	NA	NA	NA	8/92	8.7	(3.8, 16.4)	NA	NA	NA
	Grade 3	NA	NA	NA	NA	NA	NA	0/92	0	(0, 3.9)	NA	NA	NA
Injection Site Swelling	Any	NA	NA	NA	NA	NA	NA	11/92	12.0	(6.1, 20.4)	NA	NA	NA
	Grade 1	NA	NA	NA	NA	NA	NA	8/92	8.7	(3.8, 16.4)	NA	NA	NA
	Grade 2	NA	NA	NA	NA	NA	NA	3/92	3.3	(0.7, 9.2)	NA	NA	NA
	Grade 3	NA	NA	NA	NA	NA	NA	0/92	0	(0, 3.9)	NA	NA	NA
4CMenB													
Injection Site Pain	Any	NA	NA	NA	NA	NA	NA	NA	NA	NA	71/92	77.2	(67.2, 85.3)
	Grade 1	NA	NA	NA	NA	NA	NA	NA	NA	NA	27/92	29.3	(20.3, 39.8)

	Grade 2	NA	NA	NA	NA	NA	NA	NA	NA	NA	34/92	37.0	(27.1, 47.7)
	Grade 3	NA	NA	NA	NA	NA	NA	NA	NA	NA	10/92	10.9	(5.3, 19.1)
Injection Site Erythema	Any	NA	NA	NA	NA	NA	NA	NA	NA	NA	13/92	14.1	(7.7, 23.0)
	Grade 1	NA	NA	NA	NA	NA	NA	NA	NA	NA	7/92	7.6	(3.1, 15.1)
	Grade 2	NA	NA	NA	NA	NA	NA	NA	NA	NA	5/92	5.4	(1.8, 12.2)
	Grade 3	NA	NA	NA	NA	NA	NA	NA	NA	NA	1/92	1.1	(0, 5.9)
Injection Site Swelling	Any	NA	NA	NA	NA	NA	NA	NA	NA	NA	14/92	15.2	(8.6, 24.2)
	Grade 1	NA	NA	NA	NA	NA	NA	NA	NA	NA	9/92	9.8	(4.6, 17.8)
	Grade 2	NA	NA	NA	NA	NA	NA	NA	NA	NA	3/92	3.3	(0.7, 9.2)
	Grade 3	NA	NA	NA	NA	NA	NA	NA	NA	NA	2/92	2.2	(0.3, 7.6)

CI, confidence interval; M, number of subjects with available data for the relevant endpoint; n, number of subjects experiencing the endpoint listed; N, number of subjects in Safety Analysis Set. Percentages are based on M.

Group 1, MenACYW-TT primed: MenACYW-TT booster; Group 2; MCV4-CRM primed: MenACYW-TT booster; Group 3, MenACYW-TT booster +MenB-T; Group 4, MenACYW-TT booster +4CMenB

Table S5: Solicited systemic reactions after vaccine injection, by maximum intensity during the solicited period (safety analysis set)

		Group 1 (N=186)			Group 2 (N=184)			Group 3 (N=93)			Group 4 (N=92)		
**Subjects experiencing at least one:**	**Maximum Intensity**	**n/M**	**%**	**(95% CI)**	**n/M**	**%**	**(95% CI)**	**n/M**	**%**	**(95% CI)**	**n/M**	**%**	**(95% CI)**
Fever	Any	0/185	0	(0, 2.0)	4/182	2.2	(0.6, 5.5)	1/92	1.1	(0, 5.9)	4/91	4.4	(1.2, 10.9)
	Grade 1	0/185	0	(0, 2.0)	3/182	1.6	(0.3, 4.7)	1/92	1.1	(0, 5.9)	4/91	4.4	(1.2, 10.9)
	Grade 2	0/185	0	(0, 2.0)	0/182	0	(0, 2.0)	0/92	0	(0, 3.9)	0/91	0	(0, 4.0)
	Grade 3	0/185	0	(0, 2.0)	1/182	0.5	(0, 3.0)	0/92	0	(0, 3.9)	0/91	0	(0, 4.0)
Headache	Any	67/186	36.0	(29.1, 43.4)	64/184	34.8	(27.9, 42.1)	39/92	42.4	(32.1, 53.1)	36/92	39.1	(29.1, 49.9)
	Grade 1	50/186	26.9	(20.7, 33.9)	46/184	25.0	(18.9, 31.9)	22/92	23.9	(15.6, 33.9)	20/92	21.7	(13.8, 31.6)
	Grade 2	15/186	8.1	(4.6, 13.0)	15/184	8.2	(4.6,13.1)	13/92	14.1	(7.7, 23.0)	14/92	15.2	(8.6, 24.2)
	Grade 3	2/186	1.1	(0.1, 3.8)	3/184	1.6	(0.3, 4.7)	4/92	4.3	(1.2, 10.8)	2/92	2.2	(0.3, 7.6)
Malaise	Any	50/186	26.9	(20.7, 33.9)	47/184	25.5	(19.4, 32.5)	36/92	39.1	(29.1, 49.9)	37/92	40.2	(30.1, 51.0)
	Grade 1	36/186	19.4	(13.9, 25.8)	28/184	15.2	(10.4, 21.2)	16/92	17.4	(10.3, 26.7)	25/92	27.2	(18.4, 37.4)
	Grade 2	10/186	5.4	(2.6, 9.7)	15/184	8.2	(4.6, 13.1)	15/92	16.3	(9.4, 25.5)	9/92	9.8	(4.6, 17.8)
	Grade 3	4/186	2.2	(0.6, 5.4)	4/184	2.2	(0.6, 5.5)	5/92	5.4	(1.8, 12.2)	3/92	3.3	(0.7, 9.2)
Myalgia	Any	61/186	32.8	(26.1, 40.0)	64/184	34.8	(27.9, 42.1)	60/92	65.2	(54.6, 74.9)	58/92	63.0	(52.3, 72.9)
	Grade 1	43/186	23.1	(17.3, 29.8)	54/184	29.3	(22.9, 36.5)	33/92	35.9	(26.1, 46.5)	32/92	34.8	(25.1, 45.4)
	Grade 2	15/186	8.1	(4.6, 13.0)	8/184	4.3	(1.9, 8.4)	20/92	21.7	(13.8, 31.6)	22/92	23.9	(15.6, 33.9)
	Grade 3	3/186	1.6	(0.3, 4.6)	2/184	1.1	(0.1, 3.9)	7/92	7.6	(3.1, 15.1)	4/92	4.3	(1.2, 10.8)

CI, confidence interval; M, number of subjects with available data for the relevant endpoint; n, number of subjects experiencing the endpoint listed; N, number of subjects in Safety Analysis Set. Percentages are based on M.

Group 1, MenACYW-TT primed: MenACYW-TT booster; Group 2; MCV4-CRM primed: MenACYW-TT booster; Group 3, MenACYW-TT booster +MenB-T; Group 4, MenACYW-TT booster +4CMenB

Figure S1. Patient disposition flow chart

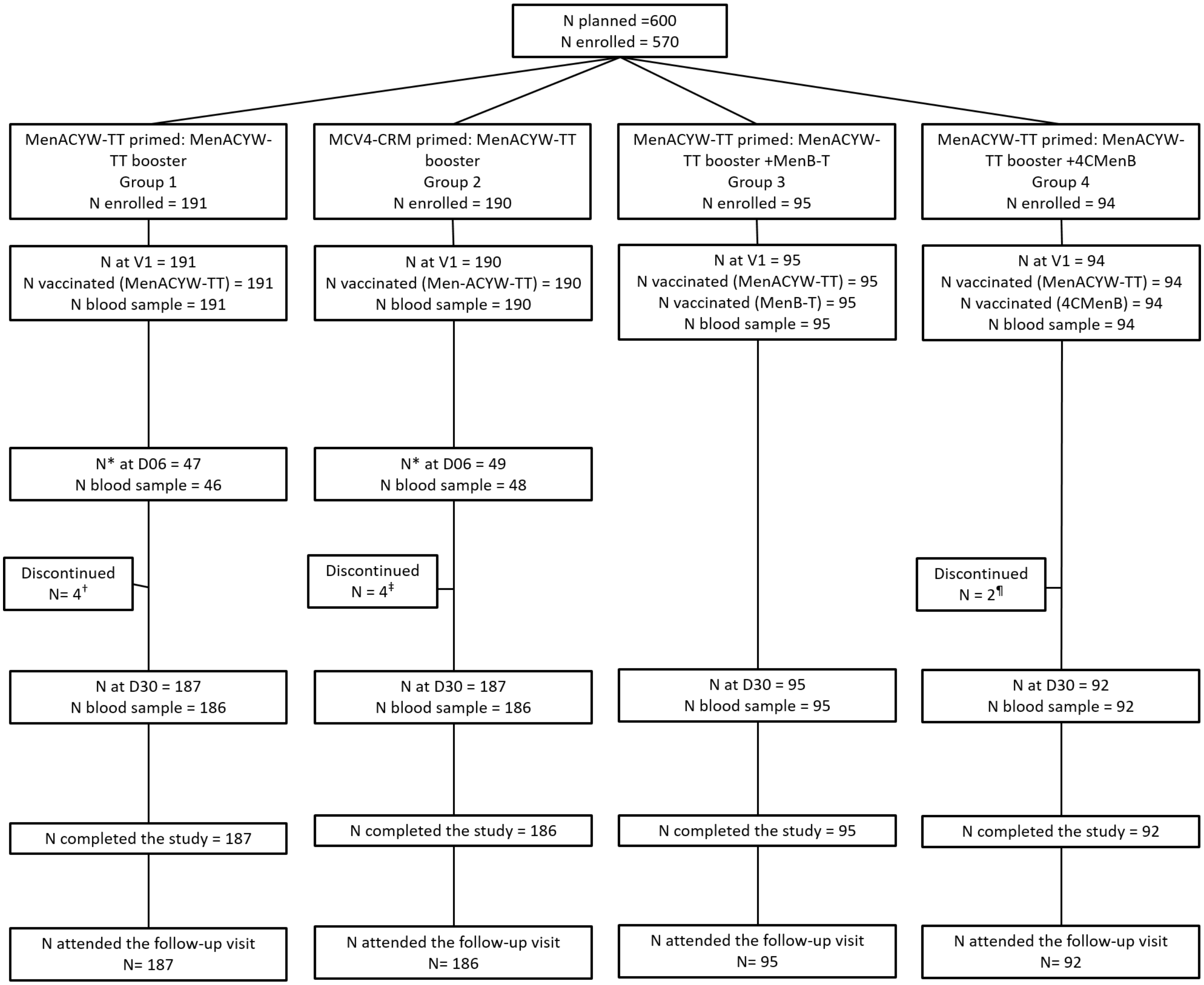

*subset cohort

† one participant was withdrawn by parent and Day 30 visit not conducted due to COVID-19, two participants were lost to follow up and Day 30 visit not conducted due to COVID-19, and one participant was enrolled in error (previously vaccinated with another meningococcal vaccine)

‡ Day 30 visit was not conducted, three of which were due to COVID-19

¶ Day 30 visit was not conducted for one participant due to parental concern about COVID-19, and one participant was enrolled in error (previously vaccinated with another meningococcal vaccine)
